# Changes in Alcohol-Specific Mortality During the COVID-19 Pandemic in 14 European Countries

**DOI:** 10.1024/0939-5911/a000841

**Published:** 2023-12-05

**Authors:** Carolin Kilian, Jürgen Rehm, Kevin Shield, Jakob Manthey

**Affiliations:** 1 Institute for Mental Health Policy Research, Centre for Addiction and Mental Health (CAMH), Toronto, Canada; 2 Campbell Family Mental Health Research Institute (CAMH), Toronto, Canada; 3 Dalla Lana School of Public Health, University of Toronto (UofT), Canada; 4 Department of Psychiatry (UofT), Toronto, Canada; 5 WHO European Region Collaborating Centre at Public Health Institute of Catalonia, Barcelona, Spain; 6 Department of Psychiatry and Psychotherapy, Centre for Interdisciplinary Addiction Research, University Medical Centre Hamburg-Eppendorf, Germany; 7 Department of Psychiatry, Medical Faculty, University of Leipzig, Germany

**Keywords:** alcohol burden, alcohol sales, substance use, public health crisis, public health, Alkoholmortalität, Krankheitslast, Substanzkonsum, Gesundheitskrise, Public Health

## Abstract

**Aim:**

Exploring trends in 1) alcohol-specific mortality and 2) alcohol sales in European countries in the years before and during the COVID-19 pandemic.

**Method:**

Complete data on alcohol-specific mortality and alcohol sales were obtained for 14 European countries (13 EU countries and UK) for the years 2010 to 2020, with six countries having mortality data available up to 2021. Age-standardised mortality rates were calculated and descriptive statistics used.

**Results:**

When compared to 2019, alcohol-specific mortality rates in 2020 increased by 7.7 % and 8.2 % for women and men, respectively. Increases in alcohol-specific mortality were seen in the majority of countries and continued in 2021. In contrast, alcohol sales declined by an average of 5.0 %.

**Conclusion:**

Despite a drop in alcohol consumption, more people died due to alcohol-specific causes during the COVID-19 pandemic in Europe.

## Zusammenfassung

**Zielsetzung:**

Beschreibung der Trendverläufe der 1) alkoholspezifischen Mortalität und 2) des Alkoholverbrauchs in europäischen Ländern vor und während der COVID-19-Pandemie.

**Methodik:**

Vollständige Daten zur alkoholspezifischen Mortalität sowie zum Alkoholverbrauch zwischen 2010 und 2020 waren für 14 Europäische Länder (13 EU-Länder und Großbritannien) verfügbar; Mortalitätsdaten aus 2021 für sechs Länder. Für die Auswertung wurden altersstandardisierte Mortalitätsraten und deskriptive Statistiken berechnet.

**Ergebnisse:**

Die alkoholspezifische Mortalitätsrate stieg 2020 ggü. dem Vorjahr um 7.7 % bzw. 8.2 % bei Frauen und Männern an. Dieser Anstieg war in den meisten Ländern zu beobachten und setzte sich im Jahr 2021 fort. Der Alkoholverbrauch sank im Mittel um 5.0 %.

**Schlussfolgerungen:**

Trotz sinkendem Alkoholkonsums sind während der COVID-19-Pandemie in Europa mehr Personen an alkoholspezifischen Erkrankungen gestorben.

## Introduction

Despite a decline in alcohol consumption since the 2000s, Europe still has the highest adult (15 years of age and older) *per capita* consumption of alcohol globally (Manthey et al., 2019). In the European Union (EU) plus the United Kingdom (UK), alcohol causes almost 320 000 deaths annually and remains the leading risk factor for premature death among 15-to-49-year-olds (GBD 2019 Risk Factors Collaborators, 2020). Recent progress towards lowering alcohol consumption (Probst et al., 2020) and its health burden (Shield et al., 2020) has been challenged by the emergence of the COVID-19 pandemic in early 2020, as particularly hazardous alcohol users were expected to increase their drinking (Gonçalves et al., 2020; Rehm et al., 2020). Hazardous alcohol use has been defined by the World Health Organization (WHO) as a pattern of alcohol use that increases the risk of harmful physical or mental health consequences to the alcohol user or to others (Saunders et al., 2019).

In the course of the pandemic, patterns of alcohol consumption changed and about half of survey participants across Europe reported decreases or increases in their drinking (Acuff et al., 2021; Kilian, O’Donnell, et al., 2022). As expected, heavier alcohol users were more likely to increase their consumption (Rossow et al., 2021; Schmidt et al., 2021), while sales data suggest decreases in consumption levels at the population level (Anderson et al., 2022; Leifman et al., 2022; Mäkelä et al., 2021). In addition to changes in alcohol consumption, it has been observed that alcohol-specific health care services were underutilized in Germany during the pandemic. Compared to 2019, hospital discharges of acute alcohol-specific conditions, such as alcohol poisonings or intoxications, dropped by −21.4 % and −25.1 % in women and men, respectively, in 2020 and there was a decline for chronic conditions, such as alcohol use disorders and alcoholic liver disease, of about −8 % (Manthey et al., 2023).

A key indicator is the number of deaths which are wholly attributable to alcohol (i. e., alcohol-specific deaths). Data from the UK and Germany suggest that alcohol-specific mortality has increased during the pandemic, by +9.9 % to +23.7 % in the UK (Angus et al., 2023) and by +5.3 % in Germany (Kilian, Carr et al., 2022). To date, it has not been explored whether this observation can be replicated in other European countries. To close this knowledge gap, we explore trends in alcohol-specific mortality in a selection of EU countries and the UK between 2010 and 2020. We contrast these trends with data on alcohol sales to obtain a more detailed picture of patterns of change across countries.

## Methodology

### Data Sources

For this explorative study, we obtained mortality data from the WHO (World Health Organization, 2023a). We included the following ICD-10 codes, indicating causes of deaths wholly attributable to alcohol, i. e., none of these deaths would have occurred in the absence of alcohol (as available in the databank): F10*, G31.2, G62.1, G72.1, I42.6, K29.2, K70*, K85.2, K86.0, Q86.0, T51*, X45. Alcohol sales data were also sourced from the WHO (World Health Organization, 2023b), expressed as the total amount of pure alcohol produced, sold, or taxed per year for the population 15 years or older (i. e., recorded *per capita* alcohol consumption). The sales data are not adjusted for tourist or unrecorded alcohol consumption and are only available until 2020.

To calculate mortality rates, i. e., the number of deaths per 100 000 population, we used United Nations population data (Population on 01 July, by 5-year age groups, medium variant; United Nations, Department of Economic and Social Affairs, Population Division, 2022). We further applied age-standardisation to account for differences in the age distribution across locations and time, employing the European Standard Population (EU-27 and EFTA standard population; European Commission et al., 2013).

### Data Analyses

We selected those countries with consistently available mortality data between 2010 and (at least) 2020, resulting in *n* = 16 countries. Two smaller countries were excluded because of low number and temporal instability of annual deaths counts (data from 2020: Cyprus: 17 deaths; Luxembourg: 74 deaths; smallest number of deaths in other countries: 239 in Greece). Our final analytic sample therefore represent a population of 253 709 000 adults aged 15 or older residing in 14 European countries (Austria, Bulgaria, Czechia, Germany, Denmark, Spain, Estonia, Finland, Lithuania, Latvia, Netherlands, Poland, Slovenia, UK). For a subset of countries, mortality data was also available for 2021 (*n* = 6 countries).

To examine country-specific trends in the age-standardised alcohol-specific mortality rates and alcohol sales, we calculated their annual change relative to 2019. Findings are presented in tables and graphs and descriptive statistics (means, percentages) were computed in R version 4.2.3 (R Core Team, 2023). The data and R code are publicly available at the Figshare repository (Manthey & Kilian, 2023).

## Results

### Cross-National Trends

Before 2020, the annual number of alcohol-specific deaths ranged between 38 694 (year 2014) and 42 303 (year 2019) across all 14 countries. Between 2019 and 2020, the number of alcohol-specific deaths increased by 3749 deaths to a total of 46 052 (+8.9 %). This increase was about three times larger than the second largest increase between 2014 and 2015 (+1496 deaths) and about 14 times larger than the median annual change observed between 2010 and 2020 (+264 deaths). In terms of age-standardised mortality rates, we observed increases of +7.7 % and +8.2 % among women and men, respectively, in 2020 compared to 2019.

### Country-Specific Trends

**Figure 1.**
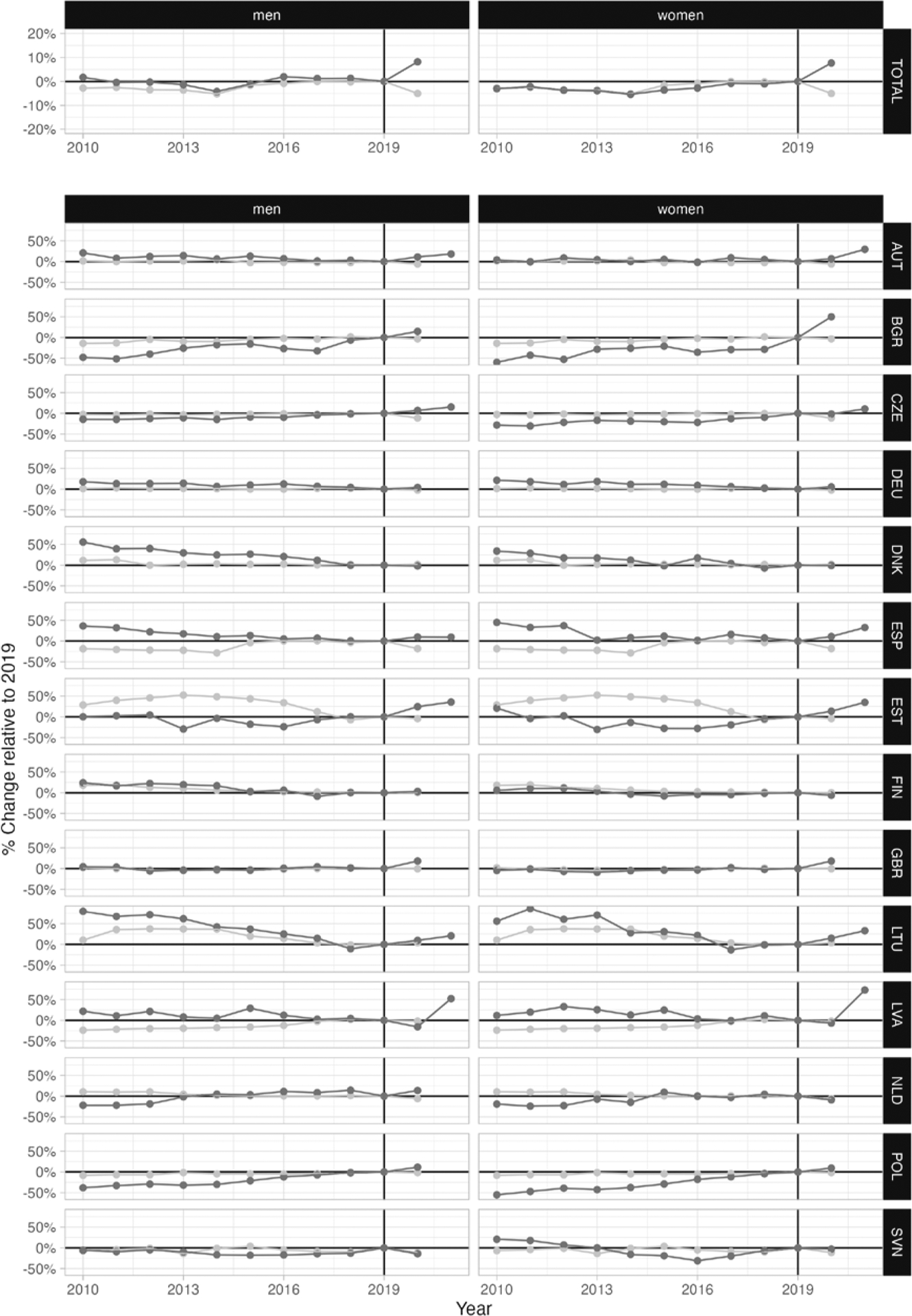
Changes in the alcohol-specific mortality rates (age-standardised; dark grey) and recorded per capita alcohol consumption (light grey) relative to 2019 (vertical line), by country. AUT: Austria, BGR: Bulgaria, CZE: Czechia, DEU: Germany, DNK: Denmark, ESP: Spain, EST: Estonia, FIN: Finland, GBR: United Kingdom, LTU: Lithuania, LVA: Latvia, NLD: Netherlands, POL: Poland, SVN: Slovenia, TOTAL: all 14 countries combined.

Sex- and country-specific data on the age-standardised alcohol-specific mortality and recorded *per capita* consumption are depicted in Table 1. In most countries, mortality rates remained largely stable between 2010 and 2019, while increasing in 2020 (see Figure 1).

Relative to 2019, the largest increases in 2020 can be observed in Bulgaria, Estonia, and UK. More moderate increases were found in Austria, Germany, Lithuania, Poland, and Spain. In Finland, Czechia, and the Netherlands, alcohol-specific mortality decreased among women but increased among men. In Denmark, Latvia, and Slovenia, decreases were observed among both women and men. In the six countries with data available from 2021, alcohol-specific mortality has increased, with the sharpest increase in Latvia (see Figure 1).

### Age-Specific Trends

**Figure 2.**
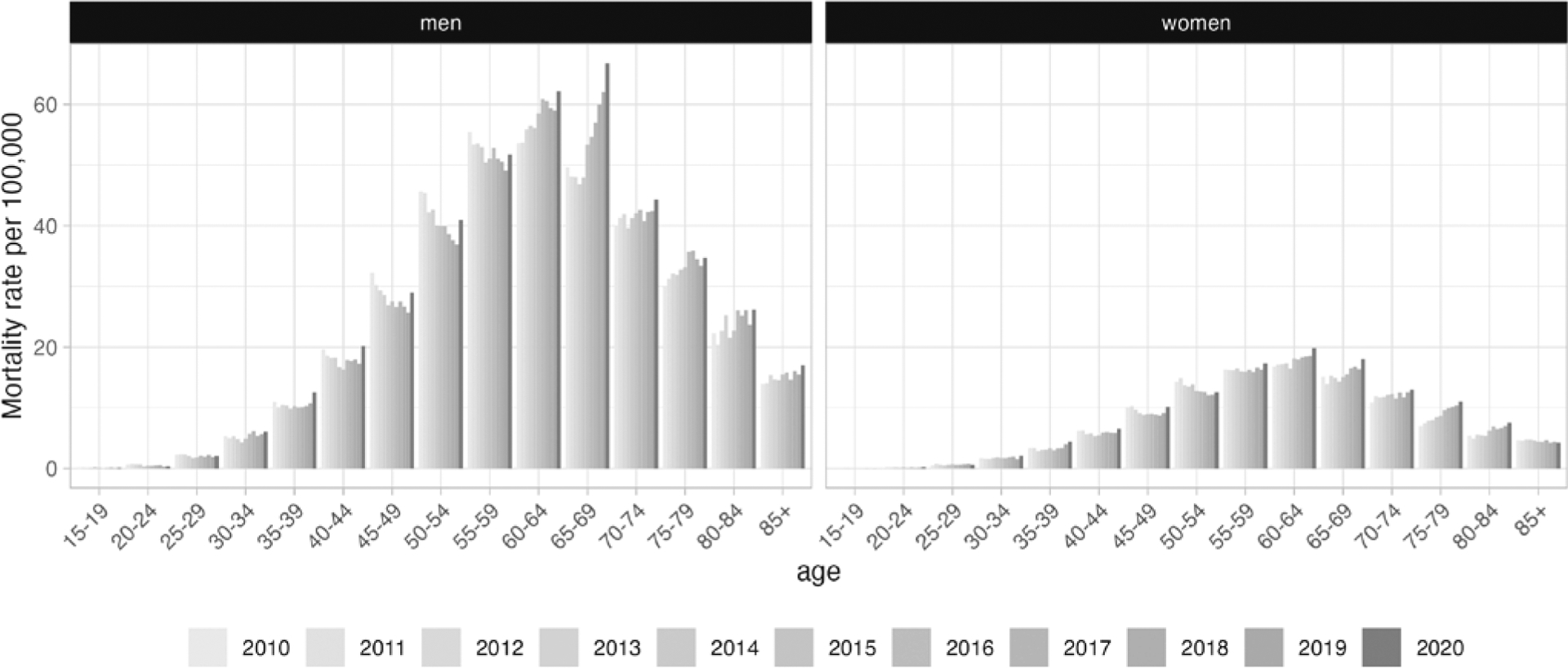
Age-specific mortality rates per 100 000 for men and women in 14 European countries between 2010 and 2020.

Figure 2 illustrates age-specific trends in mortality for all 14 countries combined. Between 2010 and 2019, alcohol-specific mortality decreased among 45-to-59-year-old women and 40-to-59-year-old men, while rates increased for older adults, except for women aged 85+, and stagnated for those below age 45 (women) and 40 (men). In 2020, an increase in mortality can be observed in all age groups, with the strongest rise among middle-aged adults. Modest changes were found among 15–29-year-olds and among women aged 85+, who generally exhibited much lower mortality rates than the other age groups.

### Trends in Alcohol Sales

Per capita consumption data suggest that consumption levels stagnated or decreased in 2020 compared to 2019 in most countries, with an average decline of −5.0 % across countries (for country results, see Table 1 and Figure 1). The most notable decline was observed in Spain, where *per capita* consumption decreased by −18.3 %.

## Discussion

In the majority of 14 European countries, alcohol-specific mortality markedly increased in 2020. In all six countries with available data, this trend continued in 2021. The pre-pandemic downward trend in alcohol-specific mortality among 45-to-54-year-old women and 40-to-59-year-old men reversed and the situation worsened in older age groups, which had already exhibited increasing mortality rates before 2020. The Baltic countries Estonia, Latvia, and Lithuania stood out with the most dramatic increases in alcohol-specific mortality. In contrast to this general upward trend in mortality in 2020, recorded *per capita* alcohol consumption decreased or stagnated in most countries.

We find that with the emergence of the COVID-19 pandemic, alcohol-specific mortality increased in several but not all European countries. As extreme situations like natural disasters or economic crises are associated with elevated levels of stress, which in turn have been shown to be associated with increases in alcohol use, especially in heavier users (De Goeij et al., 2015), increases in drinking among hazardous alcohol users as well as a rise in alcohol use disorders had been predicted at the beginning of the pandemic (Gonçalves et al., 2020; Rehm et al., 2020). As alcohol-specific deaths are the result of heavy drinking, this could explain our finding of increased mortality. Further investigations are necessary to test this mechanism.

The analyses presented are limited by multiple factors. First, causes of death may contain biases (Neuilly, 2022) and country differences may reflect regional variations in coding practices (for limitations inherent to coding alcohol-specific deaths, see Sherk et al., 2022). However, there is no obvious reason how these biases could explain our specific results on within-country trends. Second, data on the socioeconomic disparities in alcohol-specific mortality were not available. It is plausible that a disproportionate share of the excess deaths occurred among individuals with lower socioeconomic status, who generally experience higher rates of alcohol harm (Probst et al., 2021) and COVID-19 related deaths (Riou et al., 2021; Wachtler et al., 2020). Third, smoking may be an unmeasured confounder for our results, as smoking rates increased during COVID-19 (Manthey et al., 2021) and liver cirrhosis deaths wholly attributable to alcohol – in many countries the largest alcohol-specific category – may have been affected by smoking and the interaction between smoking and alcohol (Liu et al., 2008). Forth, trends in recorded *per capita* consumption may have been compensated by contrary trends in unrecorded consumption. However, many categories of unrecorded alcohol (e. g., cross-border shopping, or smuggling; Lachenmeier et al., 2021) were impeded by COVID-19 measures (Leifman et al., 2022). Thus, other categories (e. g., home-produced alcohol; surrogate alcohol) needed to increase in a way to fully compensate the decreases in major categories for unrecorded alcohol in Europe. Finally, alcohol consumption by tourists plays an important role in some countries (e. g., Spain), and decreases in alcohol sales may be in part due to the discontinuation of tourism during periods of lockdowns and border closures.

Moreover, it remains unclear to what extent impairments and between-country differences in addiction-related healthcare services contributed to the observed increases in alcohol-specific mortality in different countries. A German study suggests a notable disruption of these services in 2020 (Manthey et al., 2023) and a French study showed discrimination against people with alcohol use disorders in hospital care during COVID-19 (Schwarzinger et al., 2023). Our findings yield further support for this mechanism, as in countries with fewer restrictions and thus potentially less severe constraints on healthcare, alcohol-specific mortality did only marginally change (e. g., Denmark and Finland, which were among the few European countries without lockdowns in 2020, Hale et al., 2021).

## Conclusion

Alcohol-specific mortality increased during the COVID-19 pandemic in Europe, with some variation between countries and despite stagnant or declining alcohol sales. The reasons for this increase need to be further explored, focusing on vulnerable populations such as socioeconomically disadvantaged groups in particular, and alcohol control policies adapted.

## Figures and Tables

**Table 1.** Sex- and country-specific age-standardised alcohol-specific mortality rates and alcohol sales between 2010 and 2021.

Country	Year	Women		Men		Alcohol sales in litres pure alcohol	Change in alcohol sales relative to 2019 (%)
		Age-standardised mortality rate per 100 000	Change in mortality rate relative to 2019 (%)	Age-standardised mortality rate per 100 000	Change in mortality rate relative to 2019 (%)		
Austria	2010	6.7	.	29.8	.	12.1	.
	2015	6.8	.	27.9	.	11.6	.
	2019	6.5	.	24.6	.	11.9	.
	2020	6.9	+6.8	27.4	+11.1	11.2	−5.8
	2021	8.4	+29.5	29.1	+18.3	.	.
Bulgaria	2010	0.3	.	3.7	.	9.8	.
	2015	0.7	.	5.9	.	11.0	.
	2019	0.8	.	7.0	.	11.5	.
	2020	1.3	+49.9	8.1	+14.8	11.1	−3.4
Czechia	2010	8.8	.	29.6	.	12.7	.
	2015	9.7	.	31.4	.	12.8	.
	2019	12.2	.	34.6	.	13.0	.
	2020	12.0	−1.6	37.0	+7.0	11.6	−11.3
	2021	13.5	+10.6	39.8	+15.2	.	.
Germany	2010	10.3	.	32.5	.	11.1	.
	2015	9.5	.	30.3	.	11.0	.
	2019	8.5	.	27.6	.	11.0	.
	2020	9.0	+5.4	28.7	+4.1	10.7	−2.1
Denmark	2010	18.4	.	54.5	.	10.2	.
	2015	13.5	.	44.4	.	9.4	.
	2019	13.7	.	35.0	.	9.2	.
	2020	13.6	−1.0	34.3	−2.1	9.3	+2.0
Estonia	2010	24.2	.	76.5	.	15.0	.
	2015	14.5	.	62.8	.	16.7	.
	2019	20.0	.	76.4	.	11.7	.
	2020	22.7	+13.5	95.1	+24.5	11.1	−4.4
	2021	27.0	+34.9	103.6	+35.6	.	.
Finland	2010	16.0	.	56.9	.	9.7	.
	2015	14.0	.	47.0	.	8.5	.
	2019	15.1	.	45.8	.	8.2	.
	2020	14.2	−6.3	47.3	+3.3	8.3	+0.3
Latvia	2010	16.5	.	78.1	.	9.8	.
	2015	18.5	.	82.8	.	10.8	.
	2019	14.8	.	64.1	.	12.9	.
	2020	13.8	−6.8	54.0	−15.8	12.6	−2.0
	2021	25.6	+72.9	97.6	+52.3	.	.
Lithuania	2010	15.3	.	63.5	.	12.0	.
	2015	12.8	.	48.3	.	13.0	.
	2019	9.8	.	35.3	.	10.9	.
	2020	11.3	+14.9	38.7	+9.6	11.3	+4.0
	2021	13.0	+32.9	42.6	+20.7	.	.
Netherlands	2010	2.8	.	7.4	.	9.1	.
	2015	3.7	.	9.8	.	8.3	.
	2019	3.4	.	9.5	.	8.2	.
	2020	3.1	−9.0	10.8	+13.6	7.7	−6.0
Poland	2010	5.4	.	31.6	.	10.1	.
	2015	8.6	.	40.3	.	10.5	.
	2019	12.1	.	51.1	.	11.0	.
	2020	13.2	+9.3	56.9	+11.5	10.8	−1.6
Slovenia	2010	23.0	.	76.2	.	10.3	.
	2015	15.4	.	66.4	.	11.5	.
	2019	19.1	.	80.9	.	11.1	.
	2020	18.5	−2.9	69.2	−14.5	9.8	−11.3
Spain	2010	1.5	.	9.2	.	8.8	.
	2015	1.2	.	7.7	.	10.4	.
	2019	1.1	.	6.8	.	10.8	.
	2020	1.2	+10.9	7.4	+9.8	8.8	−18.3
	2021	1.4	+32.5	7.4	+9.4	.	.
UK	2010	8.8	.	19.9	.	10.0	.
	2015	8.9	.	18.3	.	9.5	.
	2019	9.2	.	19.1	.	9.8	.
	2020	10.9	+18.1	22.6	+18.2	9.8	−0.5
All 14 countries combined	2010	7.7	.	25.9	.	10.3	.
	2015	7.7	.	25.2	.	10.4	.
	2019	8.0	.	25.5	.	10.6	.
	2020	8.6	+7.7	27.6	+8.2	10.0	−5.0

Notes. UK: United Kingdom.
